# Dedifferentiated liposarcomas treated with immune checkpoint blockade: the MD Anderson experience

**DOI:** 10.3389/fimmu.2025.1567736

**Published:** 2025-04-30

**Authors:** Madeline B. Torres, Cheuk Hong Leung, Marianne Zoghbi, Rossana Lazcano, Davis Ingram, Khalida Wani, Emily Z. Keung, M. Alejandra Zarzour, Christopher P. Scally, Kelly K. Hunt, Anthony Conley, Andrew J. Bishop, B. Ashleigh Guadagnolo, Ahsan Farooqi, Devarati Mitra, Alison K. Yoder, Michael S. Nakazawa, Dejka Araujo, Andrew Livingston, Ravin Ratan, Shreyaskumar Patel, Vinod Ravi, Alexander J. Lazar, Christina L. Roland, Neeta Somaiah, Elise F. Nassif Haddad

**Affiliations:** ^1^ Department of Surgical Oncology, The University of Texas MD Anderson Cancer Center, Houston, TX, United States; ^2^ Department of Surgery, Cooper University Hospital, Cooper Medical School of Rowan University, Camden, NJ, United States; ^3^ Department of Biostatistics, The University of Texas MD Anderson Cancer Center, Houston, TX, United States; ^4^ Department of Sarcoma Medical Oncology, The University of Texas MD Anderson Cancer Center, Houston, TX, United States; ^5^ Department of Translational Molecular Pathology, The University of Texas MD Anderson Cancer Center, Houston, TX, United States; ^6^ Department of Radiation Oncology, The University of Texas MD Anderson Cancer Center, Houston, TX, United States; ^7^ Department of Genomic Medicine, The University of Texas MD Anderson Cancer Center, Houston, TX, United States; ^8^ Department of Investigational Cancer Therapeutics, The University of Texas MD Anderson Cancer Center, Houston, TX, United States

**Keywords:** sarcoma, dedifferentiated liposarcoma, Anti-PD1, immunotherapy, immune-checkpoint inhibitors, survival

## Abstract

**Background:**

Dedifferentiated liposarcoma (DDLPS) is one of the most common types of soft tissue sarcoma (STS) characterized by liposarcomatous differentiation and a predilection for the retroperitoneum. Despite the growing number of histology-specific immune checkpoint blockade (ICB) trials in STS, it is still difficult to identify the radiographic objective response rate (ORR) for DDLPS in the real world setting. This study aimed to evaluate the ORR and survival of patients with DDLPS treated with ICB at a single center.

**Methods:**

We conducted a retrospective study of 31 patients with pathologically confirmed DDLPS treated with ICB at MD Anderson Cancer Center between 2018 and 2023. Patient demographics, disease characteristics, treatment history, and response to ICB were analyzed. Immunohistochemical analysis was performed on tumor samples to assess immune-related markers.

**Results:**

ORR by RECIST 1.1 was 3.2% (n=1/31). Among all patients (n=31), 6% achieved partial radiographic response, while 39% had stable disease, and 55% showed progressive disease. Median progression-free survival (PFS) was 3.5 (95%CI:1.9, 4.7) months, and overall survival (OS) after ICB initiation was 19.7 (95%CI: 8.8, not reached) months. Patients without prior systemic therapy demonstrated better OS (p=0.004). Immunohistochemistry revealed no relationship between pre- or post-ICB expression of CD8, CD20, CD21 and PDL-1 and response.

**Conclusion:**

While the response to ICB in DDLPS remains limited, specific immune markers may influence treatment outcomes. CD20/21 post-ICB appear more important for prognosis. Further research is warranted to identify predictive factors for ICB efficacy in DDLPS.

## 1 Background

Dedifferentiated liposarcoma (DDLPS) is a subtype of liposarcoma, and the 4^th^ most common soft tissue sarcoma (STS), making up approximately 7.2% of all STS (1). In the 5th edition of the WHO Classification of Tumours of Soft Tissue and Bone, STS are categorized based on histogenesis and molecular characteristics into major groups, including adipocytic, fibroblastic/myofibroblastic, so-called fibrohistiocytic, smooth muscle, skeletal muscle, vascular, peripheral nerve sheath, uncertain differentiation, and undifferentiated sarcomas, reflecting advances in molecular pathology and diagnostic precision (2). Histologically, DDLPS falls under the adipocytic tumor category and tends to be moderate to high-grade with non-lipogenic, undifferentiated cells. DDLPS can arise *de novo* or from well differentiated liposarcoma (WDLPS) (3). Like WDLPS, DDLPS is characterized by 12q3-q15 amplification, associated with *MDM2, HMGA2 and CDK4* gene amplification (3, 4). Primary DDLPS has a 44% 5-year disease specific survival, with microscopic margin negative (R0) surgery, which is the mainstay of treatment for localized extremity and retroperitoneal DDLPS, with radiotherapy recommended for high grade extremity tumors (4–6). Preoperative radiotherapy is not routinely recommended at initial presentation in high grade retroperitoneal DDLPS as it appears to have limited benefit based on a multicenter randomized trial (5, 7, 8). The role of systemic chemotherapy in the localized setting is under investigation in an international multicenter randomized trial, but recommendations of an anthracycline-based therapy is the standard first line therapy for patients with unresectable, metastatic disease (5, 9–12). Despite the recommendation for systemic therapy in metastatic disease, response rates are low ranging from 12% to 24% in the first and second line setting (9, 11, 13).

Therapy with immune checkpoint blockade (ICB) has gained traction in the treatment of STS given the success in other solid tumors such as melanoma and non-small cell lung cancer (14). Furthermore, certain STS subtypes, such as undifferentiated pleomorphic sarcoma and DDLPS, exhibit features suggesting potential responsiveness to ICB, including the presence of tumor-infiltrating lymphocytes, and Programmed Death-Ligand 1(PD-L1) expression (15–17). Several non-histology specific trials have reported 5% to 20% objective response rate (ORR) in patients with metastatic STS treated with ICB (18–22). Initially, SARC028, a multicenter trial of pembrolizumab in select STS histologies showed an objective response rate (ORR) of 20% (n=2/10) for patients with DDLPS but this ORR dropped to 10% (n=4/39) in the expansion cohort with a total of 40 patients (20, 22). Specific to DDLPS, the Alliance A091401 trial reported an 8% and 14% ORR for patients treated with nivolumab and nivolumab/ipilimumab, respectively (18, 20). Furthermore, Italiano and colleagues reported a 7.3% ORR and a 54.5% non-progression rate (NPR) in a pooled analysis of several sarcoma-specific ICB trials (21). Most recently, in a non-comparative phase 2 trial of neoadjuvant ICB in patients with resectable retroperitoneal DDLPS, Roland et al. reported an 8.8% median pathologic response with 38% relapse free survival (RFS) at 24 months (23).

Despite the growing number of histology specific ICB trials in STS, it is still difficult to identify the true response rate for DDLPS outside of a clinical trial. The primary objective of this study was to determine the radiographic ORR and survival with ICB treatment in patients with DDLPS. Secondary objectives include identifying clinicopathologic and histopathologic factors that may predict response to ICB-based therapy.

## 2 Methods

### 2.1 Study design

Data were retrospectively extracted from the institutional pharmacy database of 47 patients (≥ 18 years of age) with documented DDLPS treated with ICB at the University of Texas MD Anderson Cancer Center (MDACC) between January 1, 2018, and January 1, 2023. Patients who received ICB at an outside facility are not captured by this database, even if treatment is given based on MD Anderson physician’s recommendation. Inclusion criteria required pathological confirmation of DDLPS, diagnostic confirmation with MDM2 amplification by fluorescent *in situ* hybridization (FISH), and receipt of at least one ICB dose. Patients were excluded if they received ICB as part of an ongoing clinical trial without published results or received ICB for the treatment of a second malignancy. In total, twelve patients were excluded due to enrollment in ongoing clinical trials with unpublished results and an additional four were excluded for receiving ICB as treatment for a second malignancy.

Patient demographics including age, sex, race, ethnicity, and European Cooperative Oncology Group (ECOG) performance status at the start of ICB were collected. Disease and treatment specific characteristics such as disease stage at receipt of ICB, location of primary tumor, treatment history, clinical trial participation and treatment with combination ICB were obtained. Additional data collected were duration of treatment with ICB, last known follow-up or date of death, best response to ICB treatment and time to best response. The best radiographic response in patients was determined by two methods: the Response Evaluation Criteria in Solid Tumors (RECIST 1.1) and clinical assessment as documented by the treating physician’s notes in the medical record (24, 25). ORR was defined as the percentage of patients who achieved either a complete response (CR) or a partial response (PR; at least a 30% decrease in the sum of the longest diameter (LD) of target lesions, taking as reference the baseline sum LD) as per treating physician notes or when available with precise measurements following RECIST 1.1. Data on treatment toxicity was also collected as documented by each patient’s treating physician.

### 2.2 Immunohistochemistry

All available hematoxylin and eosin (H&E) stains from a given surgical timepoint were reviewed by an experienced sarcoma pathologist to select a single formalin-fixed, paraffin-embedded sample for immunohistochemical (IHC) staining. Blocks were sectioned onto charged slides at 4-micron thickness and stained on a Leica BOND RX autostainer using the BOND Polymer Refine Detection kit. Primary antibody clones, suppliers, and dilutions used were as follows: PD-L1 (28-8, Dako, Ready-to-Use), CD8 (C8/144B, Life Technologies, 1:100), CD20 (L26, Dako, 1:1400), CD21 (2G9, Leica, 1:20). All IHC stains were evaluated by eye, except for CD8, which was scanned at 20x on an Aperio AT2 whole-slide scanner (Leica) and analyzed with HALO v3.5 image analysis platform (Indica Labs).

Assessing these specific markers provides deeper insight into the tumor microenvironment and its immune landscape, which directly impacts ICB efficacy (15). CD8+ T-cell infiltration reflects cytotoxic immune activity, with higher levels suggesting a greater likelihood of ICB response. PD-L1 expression serves as a checkpoint for immune evasion, with higher levels potentially predicting better responsiveness to PD-L1 blockade. CD20 and CD21 indicate the presence of B cells and tertiary lymphoid structures, which support adaptive immune responses. Together, these markers help define the immune phenotype of DDLPS, guiding therapeutic strategies and identifying patients who may benefit from immunotherapy.

This retrospective study was approved by the MDACC Institutional Review Board.

### 2.3 Statistical analysis

Categorical variables were reported as percentages and continuous variables as medians and interquartile ranges (IQR). Progression-free survival (PFS) was defined as the time from ICB start to progression, death, whichever occurred first, or last follow-up. Overall survival (OS) was defined as the time from start of ICB to death of any type or last follow-up (OS_ICB_) and as the time from diagnosis to death or last follow up (OS_Dx_). Landmark analysis at best response for OS and PFS was estimated by the Kaplan-Meier method (26). Log-rank test was used to compare the survival distribution by baseline characteristics. The hazard ratio was estimated in Cox proportional hazard regression model. All analyses were performed in SAS 9.4 and R 4.2.3.

## 3 Results

### 3.1 Patient characteristics

Thirty-one patients were eligible for final analysis. Most patients of the total cohort (n=28/31, 90%) received ICB as part of a clinical trial, 20 (65%) received single-agent ICB and 11 (35%) received combination ICB. Specific ICB agents are listed in **Table 1**. The patient demographic and clinical characteristics are listed in **Table 1**.

**Table 1:** Patient disease and characteristics.

Characteristic	N = 31
Age at diagnosis, median (range)	59.2 years (29.4-82.2)
Age at ICB treatment, median (range)	65.9 years (30.9-82.4)
BMI, median (range)	29.5 kg/m^2^ (18.2-41.0)
Sex, n (%)	
Female	11 (35)
Male	20 (65)
Race, n (%)	
Asian	1 (3)
Black	1 (3)
White	27 (87)
Other	2 (6)
Ethnicity, n (%)	
Hispanic/Latino	5 (17)
ECOG performance status, n (%)	
0	13 (42)
1	18 (58)
Retroperitoneal primitive tumor, n (%)	29 (94)
Number of prior lines of systemic therapy, n (%)	
0	13 (42)
1-7	18 (58)
Number of surgical resections, n (%)	
0-1	9 (29)
2-6	22 (71)
Radiation therapy*, n (%)	18 (58)
Stage at start of ICB, n (%)	
Primary	5 (16)
Recurrent	15 (48)
Metastatic	11 (35)
ICB as part of clinical trial, n (%)	28 (90)
Single agent ICB, n (%)	10 (32)
Atezolizumab	1 (3)
Nivolumab	6 (19)
Pembrolizumab	3 (10)
Combination ICB	21 (68)
Nivolumab + Ipilimumab	8 (25)
Durvalumab + Tremelimumab	3 (10)
Nivolumab + trial agent	3 (10)
Pembrolizumab + trial agent	7 (23)
Best response to ICB, n (%)	
PR	2 (6)
SD	12 (39)
PD	17 (55)
Median duration of treatment, months (range)	
PR	UC (0.9-4.2)
SD	1.4 (1.1-42.7)
PD	1.8 (0.9-4.7)
Median time to best response, months (range)	
PR	UC (0.4-2.1)
SD	1.3 (1.1-2.7)
PD	1.8 (1.0-4.7)
Median time from diagnosis to ICB start, months (range)	31.7 (0.8-222.3)
Median follow up, months (range)	49.8 (1.5-68.9)
Median overall survival**, months	19.7 (95% CI 8.8, NR)
Median progression-free survival, months	3.5 (95% CI: 1.9, 4.7)

BMI, body mass index; ECOG, Easter cooperative oncology group; ICB, Immune checkpoint blocker; NR, Not reached; PD, Progressive disease; PR, Partial response; SD, Stable disease; UC, unable to calculate.

*Receipt of radiation therapy at any point during their treatment course.

**Median overall survival since start of ICB.

Thirteen (41.9%) patients received ICB as first-line treatment, among those 12 had primary or recurrent disease, and one had metastatic disease. Patients with primary or recurrent disease had a higher proportion of ICB as first-line treatment compared to metastatic patients (60% [n=12/20] versus 9% [n=1/11], p=0.008). Fifty-eight percent (n=18/31) of the patients received at least one line of systemic therapy prior to the start of ICB.

Of those who received systemic therapy prior to ICB (n=18), 28% (n=5) received a gemcitabine-based regimen and 67% (n=12) an anthracycline-based regimen as first-line therapy. There were 71% of patients (n=22/31) who had at least two surgical resections prior to treatment with ICB, while 26% (n=8/31) had one prior resection, and only one (3%) did not have surgery prior to the receipt of ICB. Among all patients (n=31), 58% (n=18) received radiation therapy (RT) as a component of their treatment course and 39% (n=12) received it prior to ICB. The population flow chart is represented in **Supplementary Figure S1**.

Regarding the pathologic characteristics of the tumors, due to the heterogeneity of pathological reporting over the years, the final pathology reports did not systematically mention the grade and percentage of the dedifferentiated component. However, tumor necrosis was recorded in 20 out of 31 patients in our total cohort. This information was obtained from the first confirmed DDLPS sample before any ICB treatment. In total, 7 patients had no tumor necrosis identified, 5 had less or equal than 10%, and 8 had more than 10% necrosis.

### 3.2 Treatment and response to ICB

In the whole cohort, looking at response per treating physician’s notes at time of evaluation, two (6%) patients were recorded to have a PR with duration of treatment of 1.2 and 4.2 months, 12/31 (39%) were reported to have stable disease (SD) with median duration of treatment of 1.4 months (range 1.1-42.7), and 17/31 (55%) had progressive disease (PD) with median duration of treatment of 1.8 months (range 0.9-4.7). Time to best response was 1.3 months (range 1.1-2.7) for patients with SD (**Table 1**). The short response durations mentioned above may be attributable to clinical hyper-progressive disease. The median duration of treatment was 3.8 months (range 1-42.7) in patients with SD who received ICB in the metastatic setting, and 4 patients had SD as best response in the metastatic cohort. In patients who received RT before ICB (n=12), 17% (n=2) had PR, 33% (n=4) had SD, and 50% (n=6) had PD.

**Figure 1:**
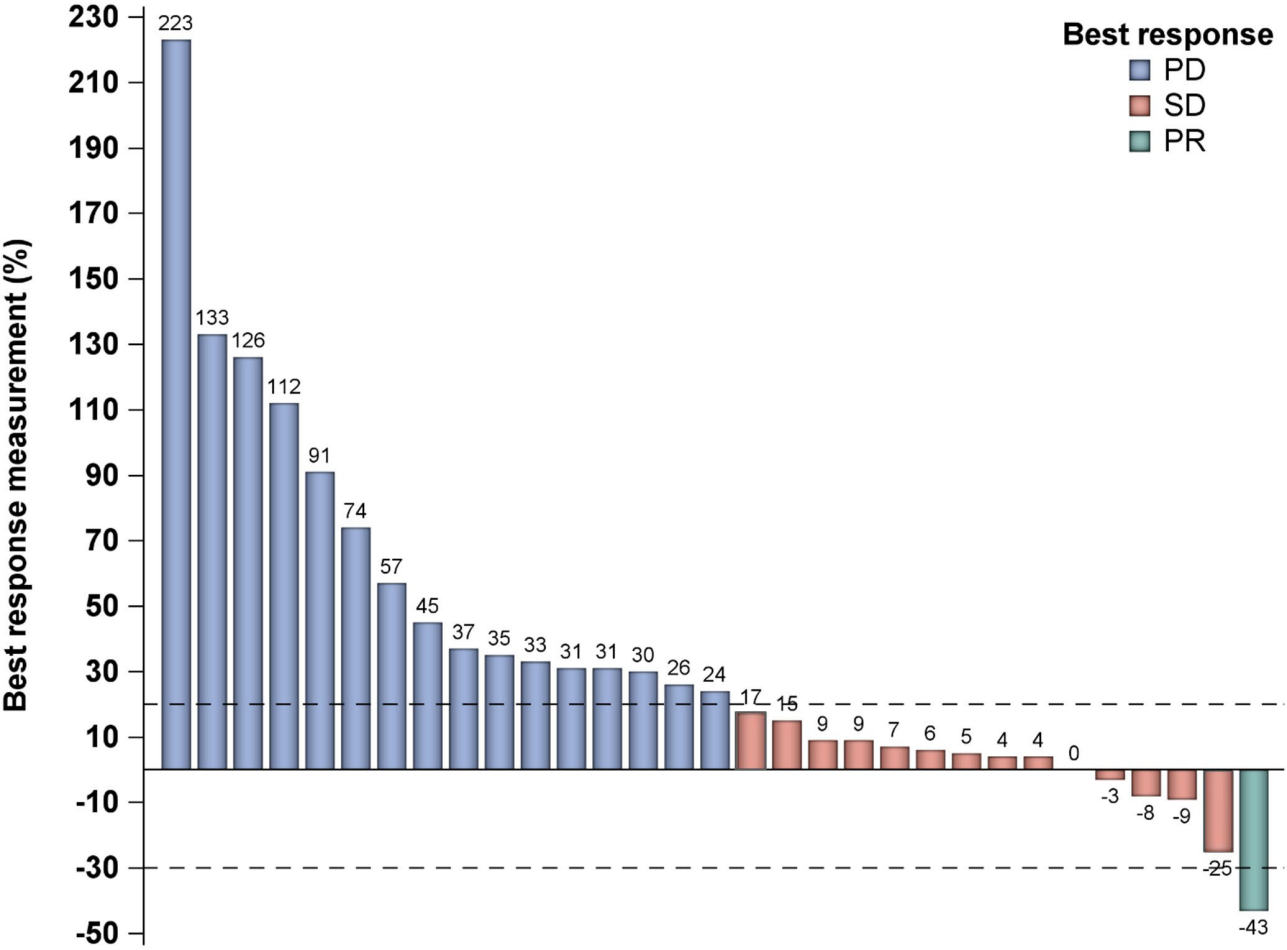
Waterfall plot demonstrating best response measurement. PD, progressive disease; PR, partial response; SD, stable disease.

Measurable responses per RECIST1.1 for the total cohort are demonstrated in the waterfall plot in **Figure 1**. The ORR per RESIST 1.1 was 3.2% (n=1/31).

### 3.3 Progression-free survival with ICB

**Figure 2:**
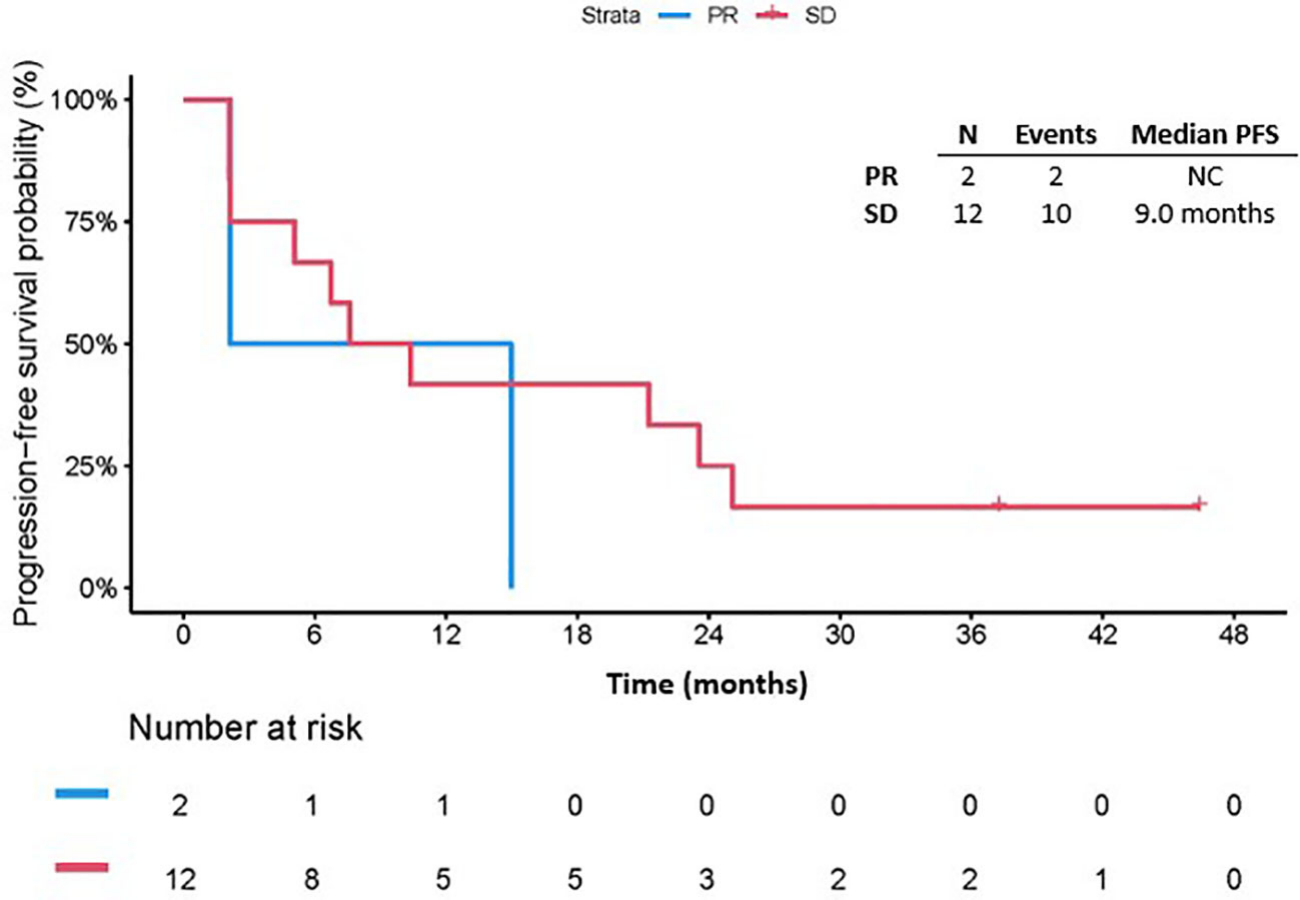
Progression free survival by number of best response. PFS, Progression free survival; PR, partial response; SD, stable disease; NC, not calculable.

The median follow-up since treatment was 49.8 months (range 1.5-68.9). Among all patients, twenty-nine (94%) had disease progression with a median PFS of 3.5 months (95% CI: 1.9, 4.7). The 3-month PFS rate was 58.1% (95% CI: 39.0, 73.1). The median PFS in patients with PR could not be calculated as there were only two patients in this cohort and their PFS since response were 4.2 months and 15.4 months. The median PFS since response in patients with SD as best response was 9.0 months (95% CI: 2.1, 25.1) (**Figure 2**). Notably, among patients who had SD as best response, 7/12 had surgery after ICB, whereas for patients who had PR as best response, 1/2 had surgery after ICB. On univariate Cox regression analysis, best response and prior receipt of systemic therapy did not appear to affect PFS (HR 0.57, 95% CI 0.12, 2.76, p = 0.485 and HR 1.07, 95% CI 0.50, 2.29, p = 0.865, respectively, **Table 2**). The risk of progression among female patients was 2.90 (95% CI: 1.21, 6.95) times the risk among male patients (p=0.017, **Table 2**). Disease stage at start of ICB (recurrent: HR 1.82, 95% CI 0.59, 5.56, p =0.295; metastatic: HR 2.24, 95% CI 0.70, 7.18, p = 0.176), number of previous surgical resections (HR 1.60, 95% CI 0.70, 3.65, p = 0.264), receipt of ICB as part of a clinical trial (HR 1.43, 95% CI 0.43, 4.76, p = 0.564) and receipt of combination ICB (HR 0.94, 95% CI 0.42, 2.10, p = 0.884) were not associated with PFS on univariate analysis (**Table 2**). In addition, ECOG performance status 0 or 1 (HR 0.76, 95% CI 0.36,1.63, p = 0.48) and retroperitoneal location of primitive tumor (HR 0.55, 95% CI 0.13,2.40, p = 0.42) did not appear to be associated with PFS (**Table 2**). The median PFS in patients who received RT before ICB was 3.4 months (95% CI: 1.4, 15.4) whereas the median PFS for those who did not receive RT before ICB was 3.8 months (95% CI: 1.3, 8.0, p=0.79; **Supplementary Figure S2**).

**Table 2:** Univariate analysis for progression free survival.

Variable	Cox Univariable HR (95% CI)	p-Value
Sex		
Male	Ref	0.017
Female	2.90 (1.21, 6.95)	
Stage at start of ICB		
Primary	Ref	0.295
Recurrent	1.82 (0.59, 5.56)	0.176
Metastatic	2.24 (0.70, 7.18)	
Retroperitoneal primitive tumor		
No	Ref	
Yes	0.55 (0.13, 2.40)	0.428
ECOG performance status		
0	Ref	
1	0.76 (0.36, 1.63)	0.483
Number of prior lines of systemic therapy		
0	Ref	
1-7	1.07 (0.50, 2.29)	0.865
Number of surgical resections		
0-1	Ref	
2-6	1.60 (0.70, 3.65)	0.264
Combination ICB		
No	Ref	
Yes	0.94 (0.42, 2.10)	0.884
ICB as part of a clinical trial		
No	Ref	
Yes	1.43 (0.43, 4.76)	0.564
Best response*		
PR	Ref	
SD	0.57 (0.12, 2.76)	0.485

CI, Confidence interval; ECOG PS, Easter cooperative oncology group performance status prior to start of ICB; HR, Hazard ratio; ICB, Immune checkpoint blocker; Ref, Reference.

*Landmark analysis at best response.

Eight out of 17 patients with PD experienced clinical rapid progression, with PD at or prior to first planned imaging evaluation. Of these 8 patients, only 2 had received prior lines of systemic therapy along with surgery and radiation. Both patients had undergone 2 prior lines of systemic therapy, with their last line before ICB being Gemcitabine and Docetaxel for 47 and 27 days, compared to duration on ICB of 5 weeks for both patients. Therefore, none of the patients in this cohort met criteria for hyperprogressive disease. Five patients had only surgery before ICB, and one patient did not receive any treatment prior to ICB.

### 3.4 Overall survival with ICB

**Figure 3:**
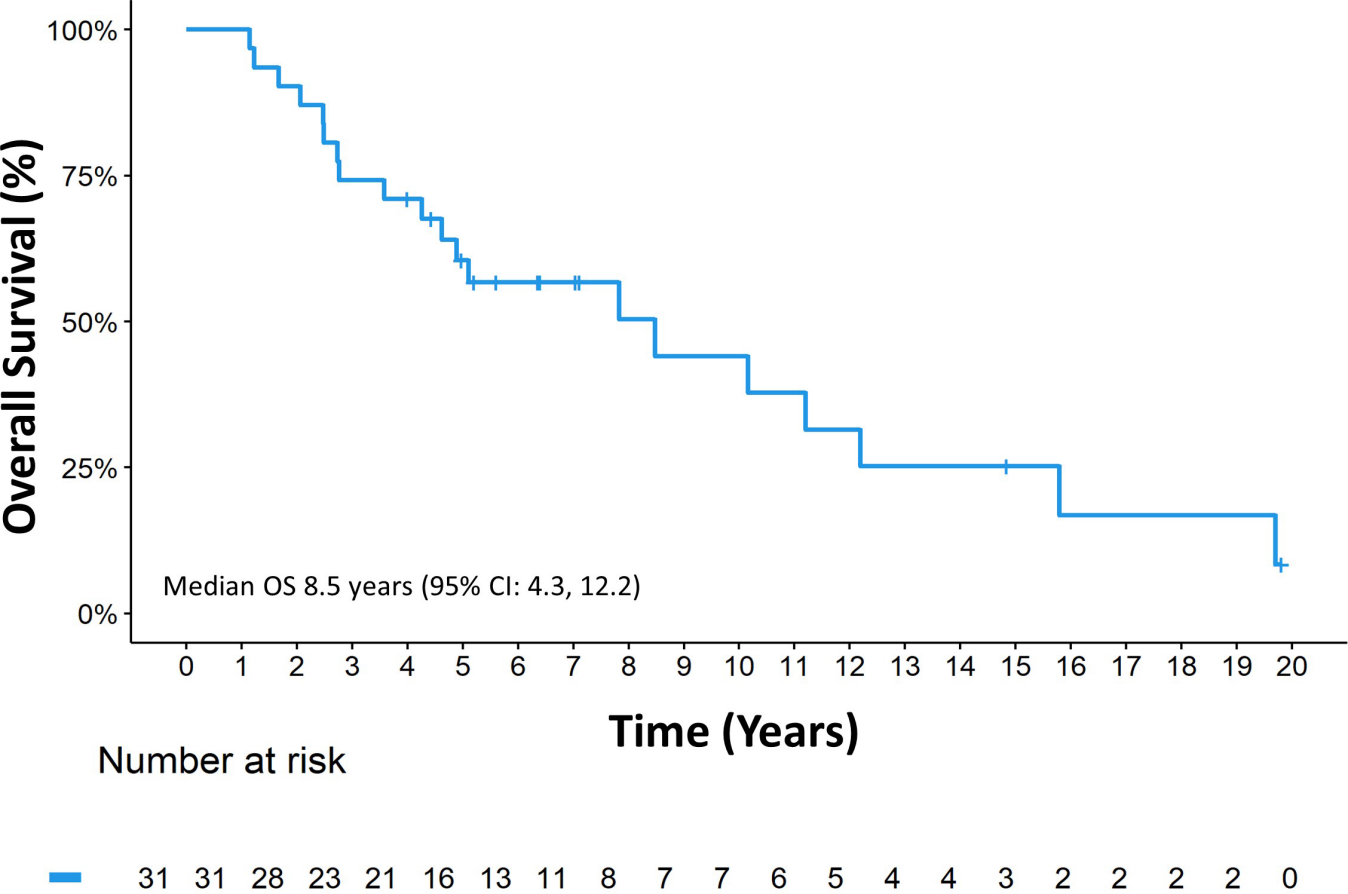
Overall survival since diagnosis.

The median follow-up since diagnosis was 14.8 years (range 1.1, 19.8), the median OS_Dx_ was 8.5 years (95% CI: 4.3, 12.2) (**Figure 3**).

**Figure 4:**
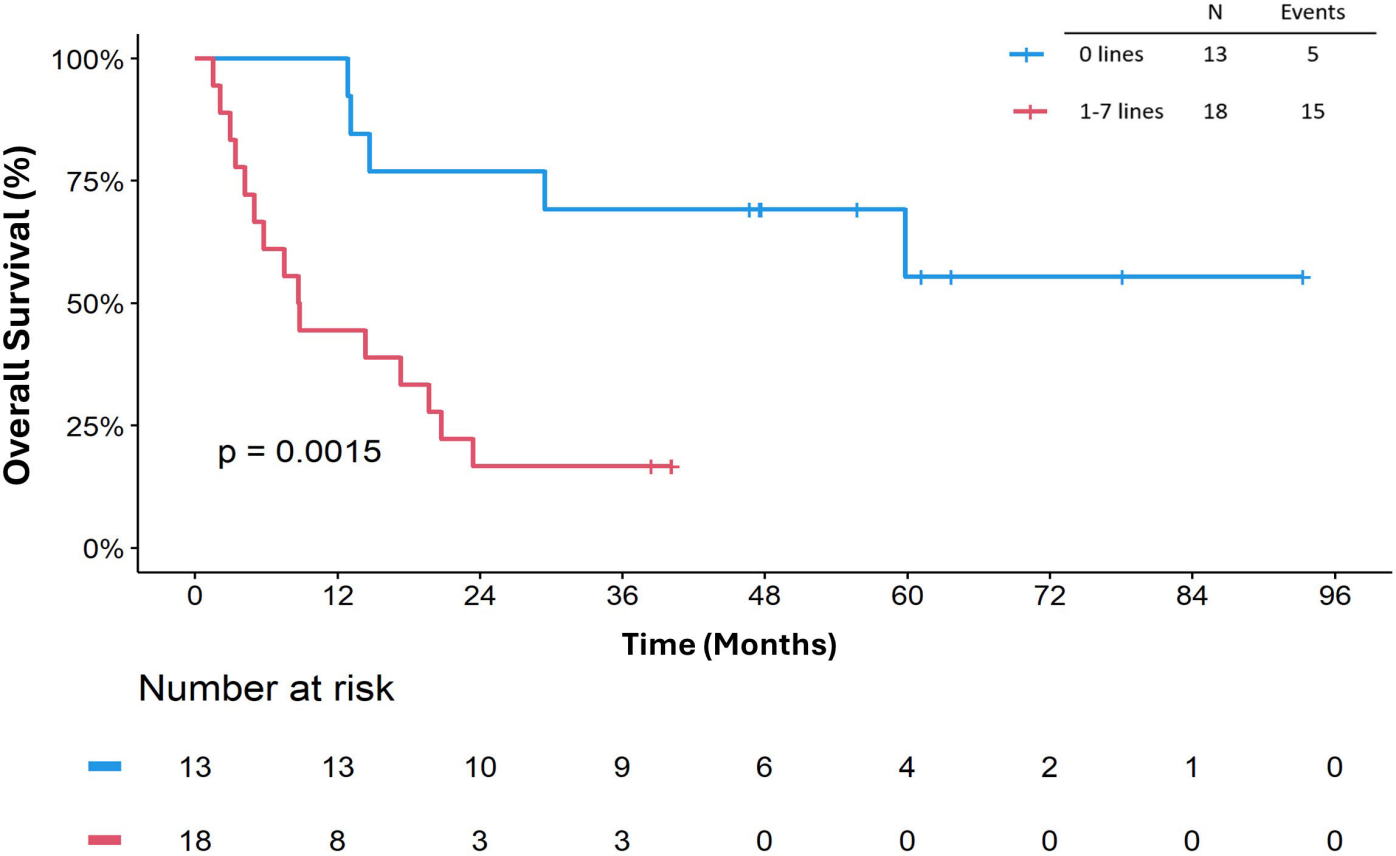
Overall survival (OS_ICB_) by number of prior lines of systemic therapy. OS_ICB_ is defined as start from immune checkpoint blocker to death or last follow-up.

The median OS_ICB_ was 19.7 months (95% CI: 8.8, not reached). Receipt of ICB prior to any systemic therapy appeared to impact OS_ICB_ (p=0.0015) (**Figure 4**). In univariate Cox regression analysis for OS_ICB_, patients who received at least one line of systemic therapy prior to ICB was associated with decreased survival, HR 5.15 (95% CI 1.68, 15.8. p = 0.004, **Table 3**) compared to patients who had no previous systemic therapy prior to ICB. Furthermore, there was no difference in OS_Dx_ between patients who received ICB as their first line of treatment versus later lines (p=0.86, **Supplementary Figure S3**).

**Table 3:** Univariable Cox proportional hazard regression for overall survival.

Variable	Cox Univariable HR (95% CI)	p-Value
Sex		
Male	Ref	0.079
Female	2.23 (0.91, 5.45)	
Stage at start of ICB		
Primary/Recurrent	Ref	
Metastatic	3.09 (1.27, 7.48)	0.013
Retroperitoneal primitive tumor		
No	Ref	
Yes	0.41 (0.09, 1.79)	0.235
ECOG performance status		
0	Ref	
1	1.96 (0.78, 4.94)	0.152
Number of prior lines of systemic therapy		
0	Ref	
1-7	5.15 (1.68, 15.8)	0.004
Number of surgical resections		
0-1	Ref	
2-6	0.76 (0.29, 2.01)	0.584
Combination ICB		
No	Ref	
Yes	0.68 (0.26, 1.78)	0.436
ICB as part of a clinical trial		
No	Ref	
Yes	0.71 (0.16, 3.10)	0.648
Best response		
PR	Ref	
SD	0.96 (0.12, 7.84)	0.971
PD	1.72 (0.22, 13.3)	0.603

CI, Confidence interval; ECOG PS, Easter cooperative oncology group performance status prior to start of ICB; HR, Hazard ratio; ICB, Immune checkpoint blocker; Ref, Reference.

**Figure 5:**
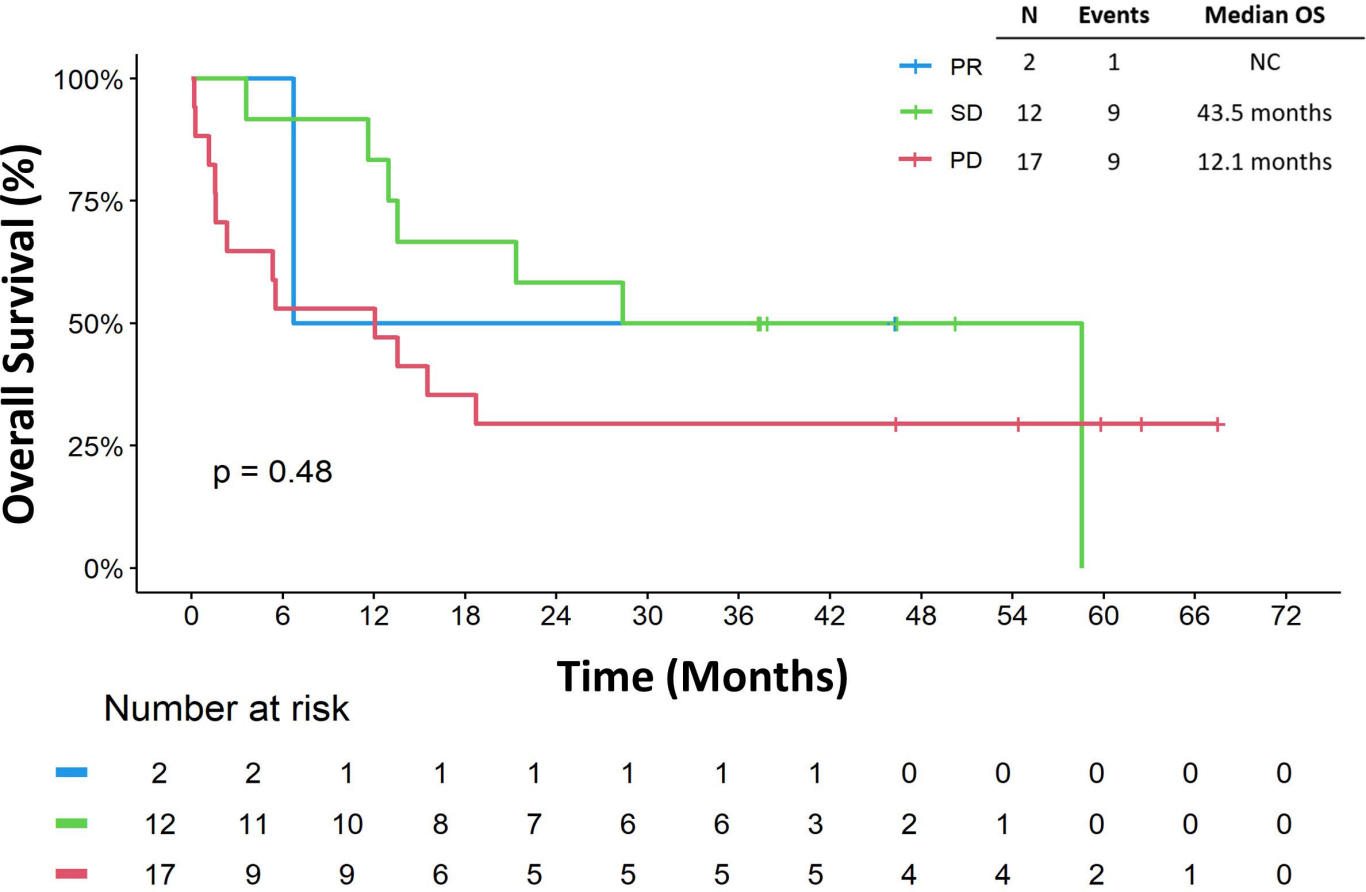
Overall survival by best response. Overall survival is defined as start from immune checkpoint blocker to death or last follow-up. NC, not calculable; OS, overall survival; PD, progressive disease; PR, partial response; SD, stable disease.

The risk of death among metastatic patients was 3.09 (95% CI: 1.27 7.48) times the risk of death among primary/recurrent patients (p = 0.013, **Table 3**). Sex (HR. 2.23, 95% CI 0.91, 5.45, p = 0.079), number of surgical resections (HR 0.76, 95% CI 0.29, 2.01, p = 0.584), receipt of ICB as part of a clinical trial (HR 0.71, 95% CI 0.16, 3.10, p = 0.648), and receipt of combination ICB (HR 0.68, 95% CI 0.26, 1.78, p = 0.436) did not appear to impact OS_ICB_ (**Table 3**). In addition, ECOG performance status 0 or 1 (HR 1.96, 95% CI 0.78, 4.94, p = 0.15) and retroperitoneal location of primitive tumor (HR 0.41, 95% CI 0.09, 1.79, p = 0.23) were not associated with OS_ICB_ (**Table 3**). Furthermore, best response to ICB did not appear to be associated with OS_ICB_ (p=0.48; **Figure 5**). When we stratified OS_ICB_ by response, the median OS since response was 43.5 months (95% CI: 11.6, not reached) in patients with SD, and 12.1 months (95% CI: 1.6, not reached) for those with PD (**Figure 5**).

### 3.5 Toxicity

Twenty-five patients experienced treatment toxicity: 17 (54.8%) grade 1, 4 (12.9%) grade 2, and 4 (12.9%) grade 3 toxicities. No grade four toxicities were reported. Five (16.1%) patients stopped ICB treatment due to toxicity. The distribution of toxicities is reported in **Supplementary Table S1**.

### 3.6 Immunohistochemistry

A total of 19 patients had tissue available for IHC staining for CD8, CD20, CD21, and PD-L1 (**Table 4**). Representative immunohistochemistry images of tumors are available in the **Supplementary Material** (**Supplementary Figure S4**).

**Table 4:** Results of Immunohistochemistry staining for CD8, CD20, CD21, and PD-L1.

IHC stain	Pre-ICB N (%)	Post-ICB N (%)
CD 8		
< median	4 (44)	9 (50)
≥ median	5 (56)	9 (50)
CD 20		
Negative	6 (75)	8 (44)
Positive	2 (25)	10 (56)
CD 21		
Negative	8 (89)	6 (46)
Positive	1 (11)	7 (54)
PDL-1		
0%	9 (100)	8 (53)
5%	–	2 (13)
40%	–	1 (7)
85%	–	1 (7)
90%	–	1 (7)
100%	–	2 (13)

ICB, Immune checkpoint blocker; IHC, Immunohistochemistry.

Of the 19 patients with available IHC staining, nine had adequate specimens prior to ICB treatment (CD20 n = 8, CD21/CD8/PD-L1 n = 9) and 18 patients had specimens available after ICB treatment for analysis (CD8/CD20 n = 18, CD21 n= 13, PD-L1 n = 15). Among these 19 patients, 4 (21%) had primary disease, 12 (63%) had recurrent disease, and 3 (16%) had metastatic disease. Eight patients had both pre and post ICB tissue available for IHC stains. Among these patients, 50% (n=4/8) had PD as best response and 50% (n=4/8) had SD as best response. Half of these patients had one or more prior line of therapy and the other half had none. Half of these patients received ICB combination and 37.5% (n=3/8) received therapy between biospecimen collection. Due to the limited number of samples, the description below is purely numerical without statistical analyses to limit the false discovery.

Before ICB, patients without prior systemic therapy more often had higher CD8 densities (n = 3/4) compared to those previously treated (n = 2/5). CD20 was negative in 75% of patients with (n = 3/4) and without (n = 3/4) prior therapy. CD21 was absent in all tumors from previously treated patients (n = 5/5) and in 75% of untreated cases (n = 3/4). All nine tumors were PD-L1 negative pre-ICB. Tumors with CD8 densities below the cohort median had progressive disease (PD) in 75% of cases (n = 3/4), versus 20% (n = 1/5) in those with higher CD8 densities (**Table 5**).

**Table 5:** Association of stains with prior lines of treatment and ICB response.

	Pre-ICB						Post ICB					
	Prior lines of systemic therapy N (%)		p	Best response N (%)		p	Prior lines of systemic therapy N (%)		p	Best response N (%)		p
	0	1-7		PR/SD	PD		0	1-7		PR/SD	PD	
CD 8			0.524			0.206			1.00			1.00
< median	1 (25)	3 (60)		1 (20)	3 (75)		6 (46)	3 (60)		4 (44)	5 (56)	
≥ median	3 (75)	2 (40)		4 (80)	1 (25)		7 (54)	2 (40)		5 (56)	4 (44)	
CD 20												
Negative	3 (75)	3 (75)	1.00	3 (75)	3 (75)	1.00	3 (23)	5 (100)	0.007	4 (44)	4 (44)	N/A
Positive	1 (25)	1 (25)		1 (25)	1 (25)		10 (77)	0 (0)		5 (56)	5 (56)	
CD 21												
Negative	3 (75)	5 (100)	0.444	4 (80)	4 (100)	1.00	3 (30)	3 (100)	0.070	2 (4)	4 (50)	1.00
Positive	1 (25)	0 (0)		1 (20)	0 (0)		7 (70)	0 (0)		3 (60)	4 (50)	
PD-L1												
0%	4 (100)	5 (100)	N/A	5 (100)	4 (100)	N/A	4 (40)	4 (80)	0.282	4 (50)	4 (57)	1.00
>5%							6 (60)	1 (20)		4 (50)	3 (43)	

ICB, Immune checkpoint blocker; N/A, not applicable; PD, Progressive disease; PR, Partial response; SD, Stable disease.

Post-ICB, CD20 and CD21 were expressed in 56% (n = 10/18) and 54% (n = 7/13) of tumors, respectively, and PD-L1 ≥ 5% was observed in 47% (n = 7/15) of patients. Among patients initially negative for PD-L1, 50% (n = 4/8) became positive after ICB. CD20 was newly expressed in 37.5% (n = 3/8) and lost in 12.5% (n = 1/8); CD21 was newly expressed in 25% (n = 2/8; **Table 6**).

**Table 6:** Characteristics of patients in whom pre/post ICB tissue was available for Immunohistochemistry stains.

	Number of prior lines of therapy	Combination ICB	ICB best response	Receipt of therapy between biospecimen collection	Pre-ICB			Post ICB		
					CD20	CD21	PDL-1	CD20	CD21	PDL-1
1	4	Yes	PD	Yes	–	–	0%	–	–	0%
2	3	No	PD	Yes	–	–	0%	–	–	0%
3	2	No	PD	Yes	+	–	0%	–	–	0%
4	0	No	PD	No	–	–	0%	+	+	85%
5	0	Yes	SD	No	–	–	0%	+	–	40%
6	0	Yes	SD	No	+	+	0%	+	+	5%
7	0	No	SD	No	–	–	0%	+	+	0%
8	1	Yes	SD	No	–	–	0%	–	N/A	90%

ICB, Immune checkpoint blocker; N/A, not applicable; PD, progressive disease; SD, stable disease; +, positive; -, negative.

CD20 expression post-ICB was found in 77% (n = 10/13) of patients without prior systemic therapy and in none (n = 0/5) of those with prior treatment. CD8 ≥ median post-ICB was seen in 54% (n = 7/13) without prior therapy vs. 40% (n = 2/5) with prior therapy. CD21 was expressed in 70% (n = 7/10) of untreated and 0% (n = 0/3) of previously treated patients. PD-L1 ≥ 5% post-ICB was seen in 60% (n = 6/10) of untreated and 20% (n = 1/5) of treated patients (**Table 5**).

Patients with CD21+ tumors post-ICB had numerically better OS and PFS (**Supplementary Figure S5**). In exploratory survival analyses, CD21+ was associated with lower risk of death (HR = 0.03, 95% CI: 0.001–0.72, p = 0.031), as was CD8 ≥ median post-ICB (HR = 0.08, 95% CI: 0.01–0.67, p = 0.019).

## 4 Discussion

Despite the success of immunotherapy for treatment of many solid tumors, DDLPS continues to be a challenge. In this study we report our institutional experience treating 31 patients with DDLPS with ICB. Regarding the evaluation of patients’ responses, RESIST has not been reliably associated with PFS and OS in the sarcoma field. Therefore, we have used two methods of evaluation, including the physician’s assessment, which is often different from RECIST (27–29). Strictly according to RESIST, we noted an ORR of 3.2% (n=1/31, patient was treated with nivolumab single-agent), which is lower than what was reported in SARC028 (ORR of 10%) and Alliance A091401 (ORR of 8% with nivolumab and 14% with nivolumab/ipilimumab) (20, 22, 30). Our findings suggest that the ORR to ICB in DDLPS is overall low. Additionally, we report a median PFS of 3.4 months (95% CI: 1.6, 4.2) and a median OS of 20 months, similar to what has been previously reported (17, 18, 20, 22, 31).

Similar to data from SARC028, Alliance A091401, and their expansion cohorts, the majority (n = 20, 65%) of patients in our cohort were treated with single-agent ICB, with limited impact on OS and RFS (17, 18, 20, 22). In the pooled analysis of phase II trials by Italiano et al.,the authors reported better ORR across all histologies with PD1/PD-L1 single agent treatment (ORR 18.7, 95% CI 2.1 - 71.6.), vs. 11.4 (95% CI 3.5 - 31.4) with combination ICB and 14 (95% CI 0.5 - 84.2.) in those treated with combination ICB with non-immunological (21). The reported ORR for patients with DDLPS with single and combination ICB was 7.3 (95% CI 1.2 - 33.7) similar to our report (21).

Anthracycline-based chemotherapy remains the standard-of-care first-line therapy for DDLPS, with an ORR around 26% and in some series as high as 40% reported (13, 32, 33). Gemcitabine-based chemotherapy (34–36) has a PFS and OS of 9.2 and 18.8 months, respectively (37). Later lines of therapy are generally reported to have around 3 months PFS with ORR of around 10% (38–40), and thus ICB in DDLPS remains a third-line and beyond therapeutic option, when compared with current standards. However, there is biological and clinical rational for introducing ICB in earlier lines of treatment, as it has been reported to be more effective in this setting (23, 41, 42). Our study included 42% (n=13) of chemotherapy-naïve patients, but these patients did not seem to have improved outcomes on ICB compared to the rest of the cohort.

Growing evidence indicates that the use of ICB in the neoadjuvant setting enhances the systemic T-cell response to tumor antigens (41) and promotes complex changes in the immune microenvironment (23, 43, 44). DDLPS are amongst the STS with higher immune infiltration making ICB therapy a promising strategy (44–47). In the phase 2 SARC032 trial, investigators evaluated the efficacy of adding pembrolizumab to the standard of care for STS (48). The study demonstrated a notable improvement in disease-free survival (DFS) with neoadjuvant immunotherapy. Specifically, patients with DDLPS who received neoadjuvant immunotherapy alongside the standard of care had significantly higher DFS compared to the control group, which did not receive immunotherapy (HR 0.55, 95% CI: 0.13–2.35) (48). However, this was in the specific setting of extremity DDLPS, and it remains unclear whether the benefit from ICB is the same in retroperitoneal DDLPS or extremity DDLPS. We have previously reported a trial of nivolumab +/- ipilimumab in the neoadjuvant setting for retroperitoneal DDLPS and the ORR and DFS were less encouraging (23). Thus, the biggest challenge in the field remains the identification of biomarkers of response to ICB for DDLPS.

Our results suggest that tumor specimens obtained after ICB are more informative than specimens before ICB in terms of prognosis, a finding that our group has already reported in our Durvalumab and Tremelimumab trial in the advanced setting (49). CD20 and CD21 seem to be amongst the most important for prognosis, in fact, patients whose tumors did not express CD21 post-ICB tended to have worse survival. This is very consistent with findings of tertiary lymphoid structures and intra-tumoral B-cells as important drivers of response to ICB, although the mechanism driving this response remains to be identified (50).

Limitations to our study include the single institution, retrospective study design with a small number of participants leading to bias in the analysis. Additionally, this cohort remains heterogeneous in clinical characteristics such as previous lines of therapy and number of previous surgeries. Our translational studies are also limited by the availability of samples, with most analyses lacking statistical power to be considered significant. Furthermore, while the response of DDLPS to immune ICB has been evaluated in only a few patients, our study reflects similar limitations, making it too early to standardize or draw definitive conclusions regarding ICB efficacy. In our study, we chose to highlight clinical variables and won’t be focusing on IHC variables. We did not run a Cox multivariable analysis for the molecular markers due to the small sample size. CD21 has shown promise as a surrogate marker, but its utility remains limited due to the small sample size and lack of validation. Given these challenges, reliable biomarkers should be further assessed, and PD-L1 evaluation could be considered to enhance future analyses.

## 5 Conclusions

While the response to ICB in DDLPS remains limited, specific expression of immune markers may influence treatment outcomes. B-cell markers after treatment may be biomarkers of treatment benefit but more mechanistic insights and larger datasets are needed. More collaborative efforts are needed to pool data in patients with DDLPS to assess benefit of ICB and identify reliable markers of response.

## Data Availability

The original contributions presented in the study are included in the article/**Supplementary Material**. Further inquiries can be directed to the corresponding author.
