# Supplementary material for: Dedifferentiated liposarcomas treated with immune checkpoint blockade: the MD Anderson experience
Source: Front Immunol. 2025 Apr 30;16:1567736. doi: 10.3389/fimmu.2025.1567736 (PMC12075363; doi:10.3389/fimmu.2025.1567736)
Supplement: Supplementary file 1 [file DataSheet1.pdf]

# *Supplementary Material*

## Figures

Supplementary  Figure 1. Population Flow Chart.

Supplementary Figure 2. Progression-Free Survival by Prior Radiation Therapy.

Supplementary Figure 3. Overall survival ($OS_{Dx}$) by number of prior lines of systemic therapy.

Supplementary Figure 4. Representative immunohistochemistry images of tumors (magnification 10x)  before and after immune-checkpoint blockade (ICB).

Supplementary Figure 5. Overall survival and progression-free survival curves by CD21 post-treatment with immune checkpoint blocker. Landmark analysis at post sample collection.

## Tables

Supplementary Table 1. Main Toxicities Distribution

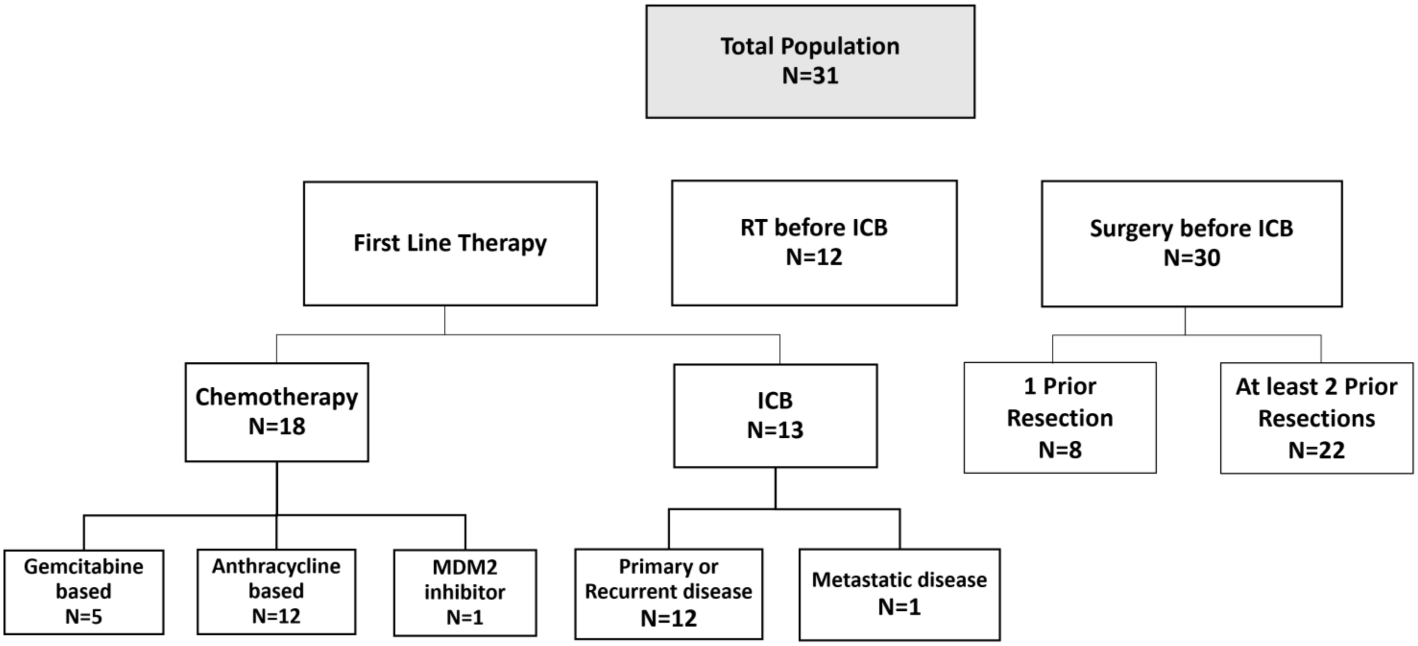


Supplementary Figure 1. Population Flow Chart. ICB = immune checkpoint blockade, RT= Radiation therapy.

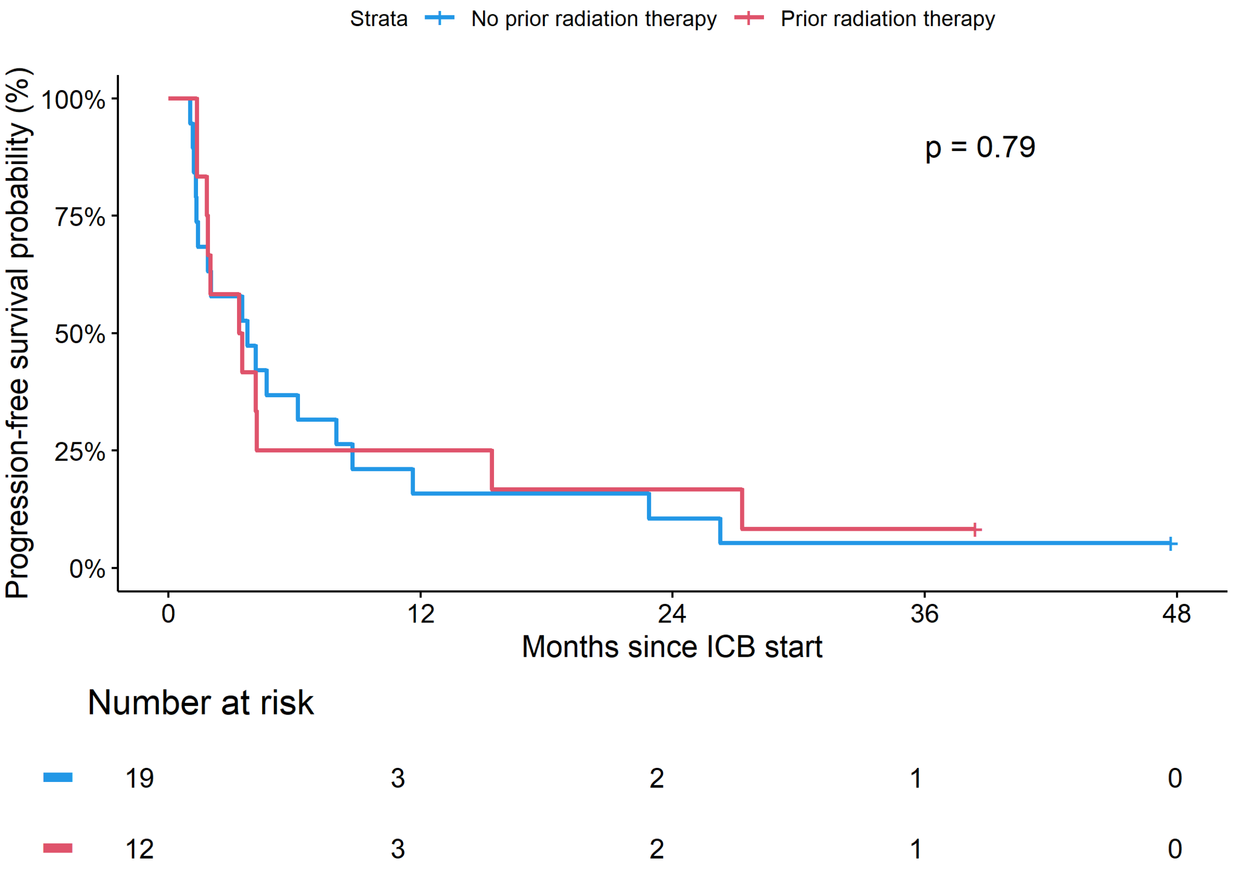


Supplementary Figure 2. Progression-Free Survival by Prior Radiation Therapy. Progression-free survival defined as start from immune checkpoint blocker (ICB) to progression or death, whichever occurred first, or last follow-up.

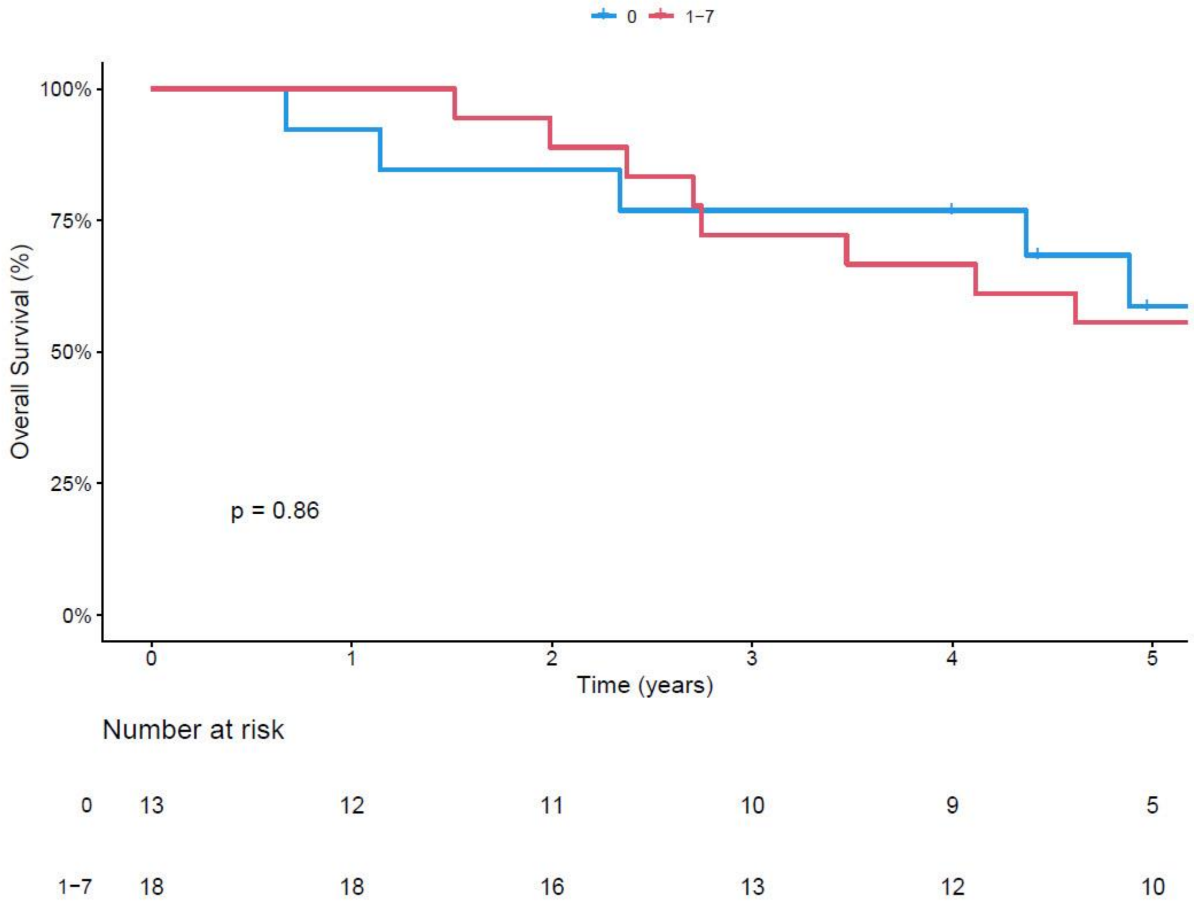


Supplementary Figure 3. Overall survival (OSDx) by number of prior lines of systemic therapy. OSDx is defined as date of diagnosis to death or last follow up.

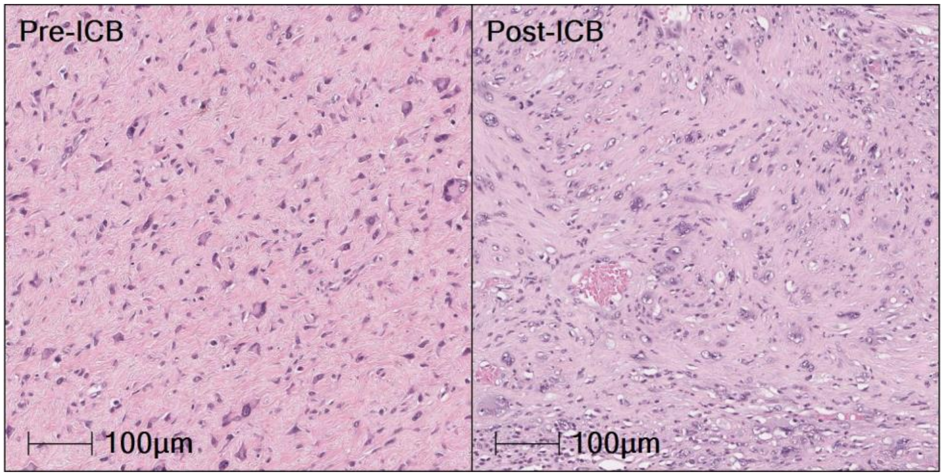


Supplementary Figure 4. Representative immunohistochemistry images of tumors (magnification 10x) before and after immune-checkpoint blockade (ICB).

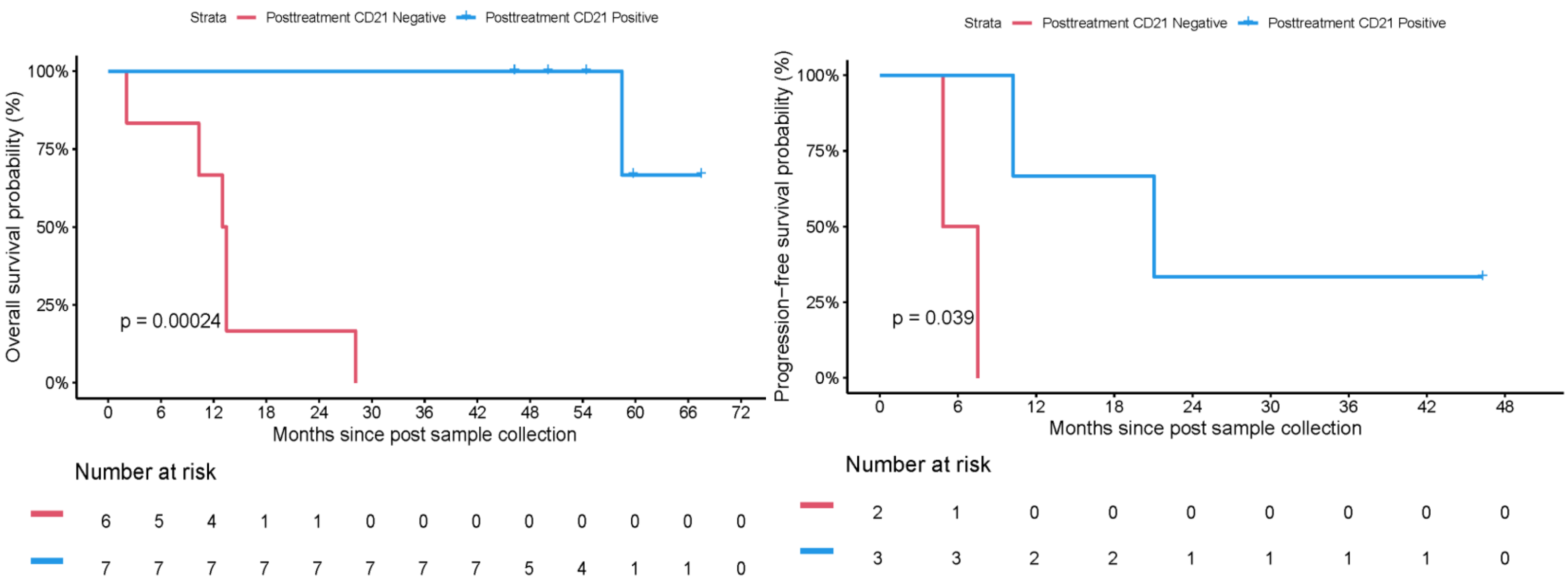


Supplementary Figure 5. Overall survival and progression-free survival curves by CD21 post-treatment with immune checkpoint blocker. Landmark analysis at post sample collection.

Supplementary Table 1. Main Toxicities Distribution

| Toxicity | Grade 1-2 N (%) | Grade 3 N (%) |
|---|---|---|
| Fatigue | 5 (16.1) | 3 (9.7) |
| Rash | 4 (12.9) | 2 (6.5) |
| Pruritis | 3 (9.7) | 1 (3.2) |
| Constipation | 3 (9.7) | 0 (0) |
| Bilateral lower extremity edema | 3 (9.7) | 1 (3.2) |
| Fever | 3 (9.7) | 0 (0) |
| Chills | 3 (9.7) | 0 (0) |
| Dry mouth | 3 (9.7) | 0 (0) |
| Anemia | 3 (9.7) | 0 (0) |
| Nausea | 2 (6.5) | 0 (0) |
| Diarrhea | 2 (6.5) | 2 (6.5) |
| Cough | 2 (6.5) | 0 (0) |
| Vomiting | 1 (3.2) | 0 (0) |
| Transaminitis | 1 (3.2) | 0 (0) |
| Immune nephritis | 1 (3.2) | 0 (0) |
| Night sweats | 1 (3.2) | 0 (0) |
| Hypoalbuminemia | 1 (3.2) | 0 (0) |
| Hypothyroidism | 1 (3.2) | 0 (0) |
| Hyperthyroidism | 1 (3.2) | 0 (0) |
| Thrombocytopenia | 1 (3.2) | 0 (0) |
| Oral dysesthesia | 1 (3.2) | 0 (0) |
| Sialorrhea | 1 (3.2) | 0 (0) |
| Altered taste | 1 (3.2) | 0 (0) |
| Dyspnea | 1 (3.2) | 0 (0) |
| Hyperglycemia | 1 (3.2) | 0 (0) |
| Hyponatremia | 0 (0) | 1 (3.2) |
| Colitis | 0 (0) | 1 (3.2) |
| Herpes simplex virus | 0 (0) | 1 (3.2) |
| Autoimmune hepatitis | 0 (0) | 1 (3.2) |
| Leukocytosis | 0 (0) | 1 (3.2) |
